# Enhancing stock timing predictions based on multimodal architecture: Leveraging large language models (LLMs) for text quality improvement

**DOI:** 10.1371/journal.pone.0326034

**Published:** 2025-06-18

**Authors:** Mingming Chen, Yifan Tang, Qi Qi, Hongyi Dai, Yi Lin, Chengxiu Ling, Tenglong Li

**Affiliations:** 1 Academy of Pharmacy, Xi’an Jiaotong-Liverpool University, Suzhou, Jiangsu, China; 2 Institute of Population Health, Faculty of Health & Life Sciences, Waterhouse Building, University of Liverpool, Liverpool, United Kingdom; 3 Faculty of Computing and Data Sciences, Boston University, Boston, Massachusetts, United States of America; University of Queensland - Saint Lucia Campus: The University of Queensland, AUSTRALIA

## Abstract

This study aims to enhance stock timing predictions by leveraging large language models (LLMs), specifically GPT-4, to filter and analyze online investor comment data. Recognizing challenges such as variable comment quality, redundancy, and authenticity issues, we propose a multimodal architecture that integrates filtered comment data with stock price dynamics and technical indicators. Using data from nine Chinese banks, we compare four filtering models and demonstrate that employing GPT-4 significantly improves financial metrics like profit-loss ratio, win rate, and excess return rate. The multimodal architecture outperforms baseline models by effectively preprocessing comment data and combining it with quantitative financial data. While focused on Chinese banks, the approach can be adapted to broader markets by modifying the prompts of large language models. Our findings highlight the potential of LLMs in financial forecasting and provide more reliable decision support for investors.

## 1. Introduction

The stock market serves as an effective channel for capital allocation within an economy, playing a crucial role in the price discovery process that is essential for maintaining the health and stability of the financial system [1–3]. The price discovery process depends on the complex interplay of various factors, including firm and industry specific features, macroeconomic environment, momentum effects, and political and geopolitical climate [4–7]. Market participants collectively engage in this intricate mechanism, ensuring the efficient operation of financial markets [8–10].

Stock timing, a price discovery mechanism where market participants identify stocks that are considered to be “mispriced” in the short or long term, offering attractive return potentials relative to the broader market [11–12]. This mechanism is the core of strategies adopted by top trading masters like Gann. However, the concept of “mispricing” can be generalized to include perceived fair market prices, which may not always align with intrinsic values. This often involves expectations of future company growth, a strategy known as “growth investing,” which sometimes overlooks current fundamentals. Furthermore, in markets with a high proportion of retail investors, like China, the opinions formed from news and fund flows can lead to mispricing, which presents opportunities for tactical stock timing [13–16].

Existing literature has shown that retail investor data can be informative for stock timing, particularly since stocks frequently discussed by investors often see price increases [17]. However, using individual investor comment data for stock timing poses several challenges. First, the quality of user comments varies broadly, with many lacking substantial contents or being biased, thereby reducing data reliability [18]. Second, comment data often contain a significant amount of redundant information that introduces noise to the downstream analysis [19–20]. Additionally, the rich sources of comment data make it more difficult to check the authenticity of comments [21]. This issue is exacerbated by the prevalence of false information and emotional comments on social media and online forums. Therefore, it is crucial to effectively preprocess these comment data to enhance stock timing effectiveness. Through systematic filtering and associated analytic architectures, we aim to address these challenges, and thus improve the profit-loss ratio.

Large language models (LLMs) like GPT-4 can weigh and reason among various categories of data to complete complex financial reasoning tasks [22–23]. The GPT-4 model can even be configured to function as an expert financial analyst, using a thought chain approach to guide the model through a logical, multi-step reasoning process that mirrors the thinking patterns of professional financial analysts [24–25]. This technology effectively provides structured data-based insights for stock selection, particularly in the complex field of finance where expert-level reasoning is critical. Additionally, contextual learning is employed to dynamically adjust the analysis based on current financial conditions and evolving market data [26]. This dual approach enables the system to deliver insights that are adaptive to volatile market conditions and investor preferences, marking a significant advancement in AI-driven investment analysis.

This study conducts several experiments to investigate the usage of online comment data in stock timing. First, we will evaluate the actual impact of individual investor comments on improving the profit-loss ratio by comparing the performance of different preprocessing filter models, so as to determine whether comment data can significantly enhance investment decision accuracy. Second, we will explore the potential value of applying large language models, particularly GPT-4, to the tasks of filtering and analyzing comment data for stock timing. Finally, we propose a multimodal architecture where stock price data and technical indicators are integrated with comment data for comprehensive analysis, and the multimodal architecture is further tested to examine whether it can significantly enhance the profit-loss ratio in stock timing. Through these experiments, we hope to identify optimal method for preprocessing and analyzing comment data in stock timing, thereby providing more reliable and effective decision support for investors.

## 2. Methods

### 2.1. Data

We use raw data from nine banks, which represent three distinct types of banking institutions in China, as shown in Table 1. Covering the period from September 26, 2018, to December 31, 2021, the dataset includes a total of 328,536 commentary posts and has the following three parts: Comments, Stock Dynamic Prices, and Stock Technical Indicators, all collected from the Guba online investor forum. Our data crawling and analysis methods comply with standard internet data usage protocols [27]. The dataset comprises:

**Table 1:** Description of Bank Names and Comment Numbers.

Bank Type	Bank Names	Number of Comments
State-Owned Banks	Agricultural Bank of China (ABC)	40863
	Bank of China (BOC)	28121
	China Construction Bank (CCB)	33466
Joint-venture Banks	Shanghai Pudong Development Bank (SPD BANK)	28873
	China Minsheng Banking Corp (CMBC)	43969
	China Merchant Bank (CMB)	52485
Local Commercial Banks	Bank of Beijing (BOB)	39217
	BANK OF Guiyang (GYB)	35032
	BANK OF Jiangsu (JSB)	26510

1. Comments: This part contains textual discussions about each bank from investors, including opinions about market trends, investment insights, and trading strategies.
2. Stock Dynamic Prices: We measure the dynamic changes in stock prices, by tracking fluctuations from historical values to current levels. The specific measurements are the opening price, closing price, highest price, lowest price, and trading volume.
3. Stock Technical Indicators: These are mathematical indicators calculated from trading data such as price and volume, designed to predict market trends and support investment decisions. Primarily used in technical analysis, these indicators help identify price patterns and market trends. In our architecture, we calculate several key technical indicators [28,29]:
4. Moving Average (MA): A trend-following indicator that smooths out price data to identify the direction of a trend over a specified period. It is used to reduce noise and capture the overall movement of stock prices.
5. Stochastic Oscillator (KDJ): A momentum indicator comparing a stock’s closing price to its price range over a specific period. It helps identify overbought and oversold conditions, providing signals for potential price reversals.
6. Moving Average Convergence Divergence (MACD): A momentum and trend-following indicator that shows the relationship between two moving averages of a stock’s price. MACD is used to identify potential buy and sell signals, as well as the strength of price movements.
7. Bollinger Bands (BOLL): A volatility indicator consisting of a moving average and two standard deviation lines (bands) above and below it. Bollinger Bands help identify periods of high or low volatility and potential price reversals.

Each of these indicators plays a crucial role in assessing the market trends and informs investment decisions by providing actionable insights into price patterns and market dynamics [30–33].

### 2.2. The filter-based model for processing commentary data

**Fig 1:**
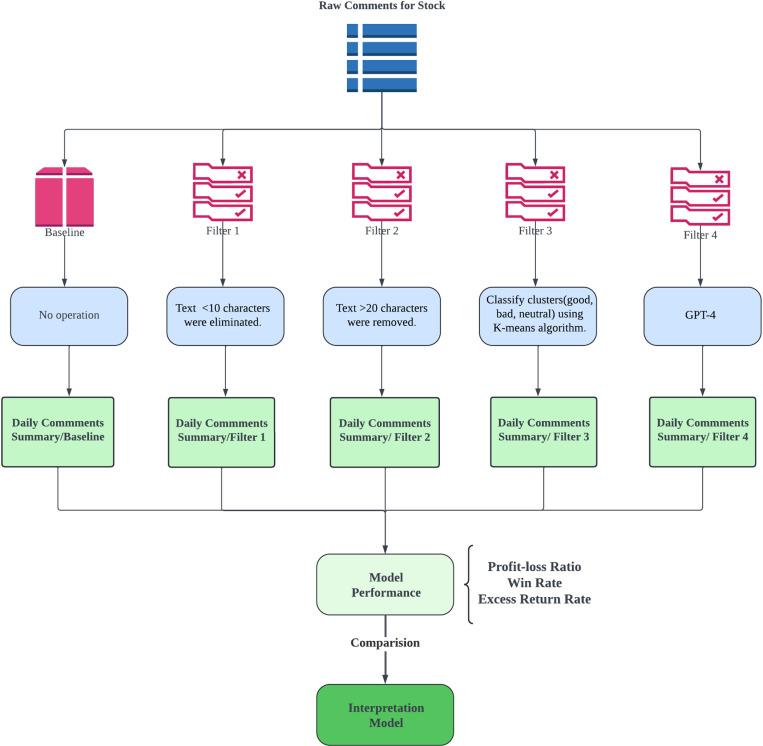
Design and Evaluation Workflow of Data Screening Filters.

Fig 1 illustrates the design of four distinct filters for the screening operation as well as a baseline model, with the goal of improving the consistency of text meaning by removing redundant data [34]. After filtering, the remaining data is merged based on the same date and random forest (RF) is used to obtain the final prediction results. The training set includes data from September 26, 2018, to December 31, 2020, and the testing set is built on data from the year 2021. The model performance is evaluated using Monte Carlo Cross-Validation (MCCV) [35], which assesses the performance through multiple rounds of random sampling and model training, and it is more comprehensive compared to traditional K-fold cross-validation. The model performance is assessed based on three metrics: profit-loss ratio, win rate, and excess return rate.

### 2.3. The multimodal architecture for optimizing excess return rate

**Fig 2:**
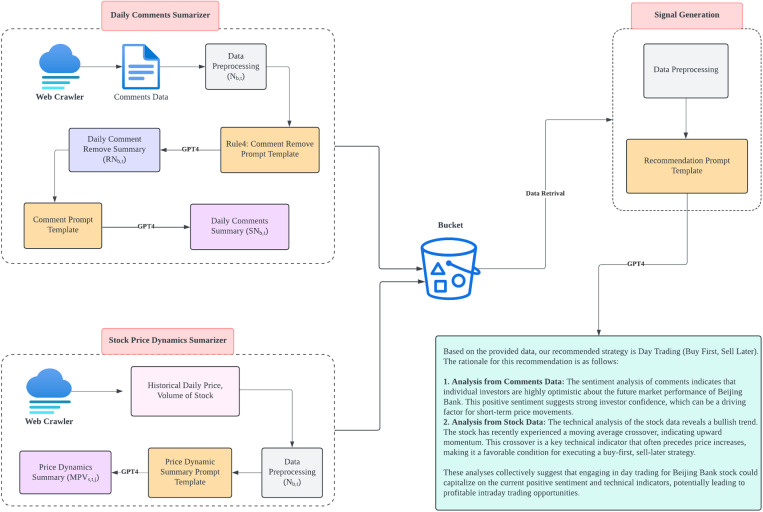
The Conceptual Framework of the multimodal architecture for optimizing the excess return rate.

With the optimal screening filter identified in the previous experiment, we proposed the multimodal architecture depicted in Fig 2 to calculate the final excess return rate. The design of this architecture is based on [36]. There are three parts of the input for this architecture: commentary data, stock prices, and stock technical indicators, designed to generate final recommendations for stock timing (i.e., buying then selling or selling then buying). The decision-making component of the architecture is responsible for synthesizing all the input information, integrating insights from commentary data, analyzing stock price trends, and evaluating technical indicators. It consolidates these data points to provide concise explanations for the corresponding stock timing decisions, such as identifying optimal entry and exit points. Each component of the architecture, including the decision-making module, was built using OpenAI’s API (the GPT-4 model) [37], utilizing zero-shot prompting and contextual learning to perform various tasks efficiently [38,39].

### 2.4. Daily comments summarizer

**Fig 3:**
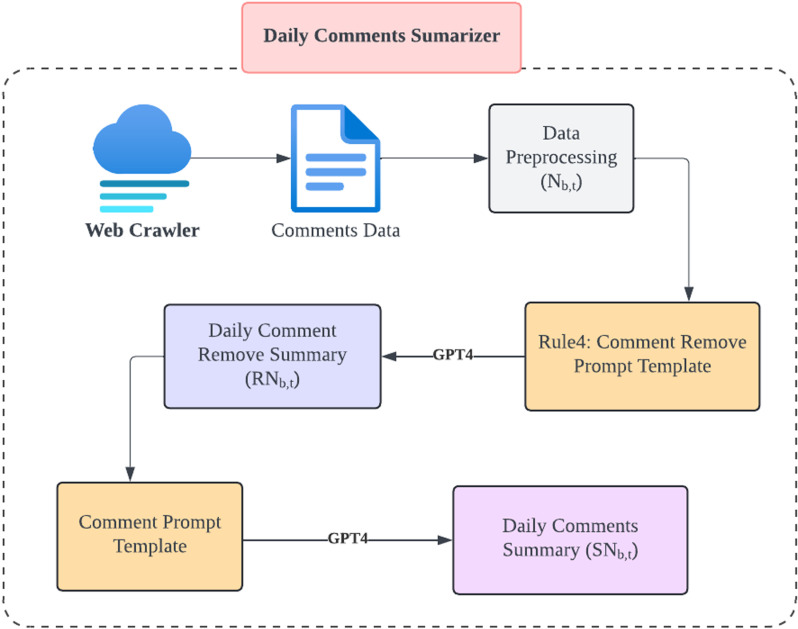
Architectural Breakdown of the Daily Comments Summarizer Module.


Individual investors’ comments about a company may have a consequential impact on market sentiment and stock prices [40]. Depending on their content, such comments may have short-term, long-term, or minimal impact, and therefore it is important to appropriately process multiple user comments on the same day. As shown in the Daily Comments Summary module in Fig 3, we use crawler technology to collect user comment data from the GuBa platform, which mainly contains opinions from individual investors on specific stocks. The company’s daily comment data is cleaned and pre-processed, in order to exclude text unrelated to stock performance, such as online advertisements and headline bait articles. GPT4 will prompt us for the daily comments ${RN}_{b,t}$ that need to be removed and output the final daily comment data ${SN}_{b,t}$, as given by the formula below.

$$\begin{array}{c} {\text{SN}}_{\text{b,t}}=⨁\text{Summarize}({\text{N}}_{\text{b,t}}-{\text{RN}}_{\text{b,t}}) \end{array}$$ (1)

For the above formula, $N_{b,t}$ represents all comment data for bank *b* on day *t* and ${RN}_{b,t}$ denotes the removed comment data for bank *b* on day *t*. The function Summarize() synthesize an updated summary, with the symbol ⨁ denotes the operation of concatenating daily comment summaries. ${SN}_{b,t}$ represents the final comment data for bank *b* on day *t*.

For example, the Daily Comments Summarizer effectively captured the changing comment narrative surrounding Bank of China on 2022-01-07 (Appendix Table 1 in S1 File). Particularly, GPT4 deleted 7 comments and retained 8 comments. Among the seven deleted comments, the first one was a bearish comment by the user and the others were neutral comments. The eight retained comments are all bullish comments, which ensures the semantic consistency of the comments on that day [41–42].

### 2.5. Stock price dynamic summarizer

**Fig 4:**
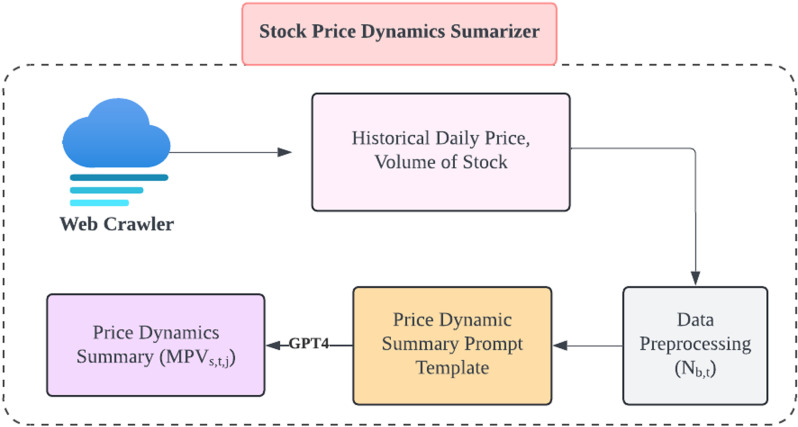
Architectural Breakdown of the Stock Price Dynamic Summarizer Module.


The stock price dynamic summarizer is a key component of the proposed architecture and is used contextualize a metric based on stock price (such as the stock technical indicators in 2.1) in Fig 4. The mathematical representation of a dynamic summary of stock prices is given by Equation 2.

$$\begin{array}{c} {\text{MPV}}_{\text{s,t,T}}={⋃}_{\text{t}=1}^{\text{T}}\text{Summarize}({\text{Metrics}}_{\text{s,t}},\;{\text{PV}}_{\text{s,t}}) \end{array}$$ (2)

where ${PV}_{s,t}$ denotes the five indicators for stock *s* on day *t*: opening price, closing price, highest price, lowest price, and trading volume. ${Metrics}_{s,t}$ represents the technical indicators for stock *s* on day *t*. The function Summarize() synthesizes an updated summary, and ${MPV}_{s,t,T}$ refers to the merged summary of stock *s* for the past *T* days (*t = 1,…,T*).

The stock price summarizer starts by analyzing a stock’s price and volume over T days. During this process, it calculates corresponding technical indicators such as MA, KDJ, MACD and BOLL. These indicators can provide insight into a stock’s performance trends and potential future movements. After calculating these metrics, the summarizer integrates the results into a comprehensive data set, which forms the final input for further analysis and decision-making, encompassing all relevant price dynamics and technical indicators to provide a robust foundation for subsequent financial evaluations and trading strategies.

### 2.6. Signal generation

**Fig 5:**
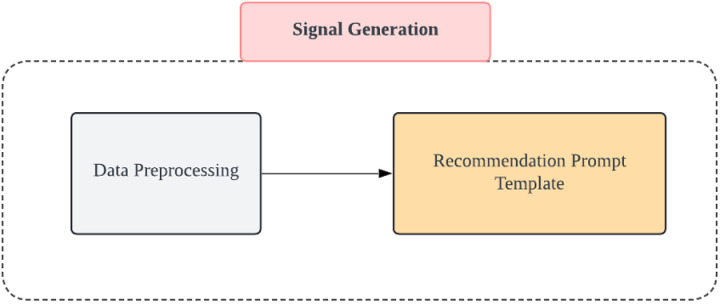
Architectural Breakdown of the Signal Generation Module.


The signal generation component is based on the output of individual user comments and stock price dynamics components, served as the final stage of the system architecture (Fig 5). Given that short-term investment decisions in stocks depend on the trend of the stock and the current public opinion environment, the decision model is expressed as follows:

$$\begin{array}{c} {\text{G}}_{\text{s}}=\text{f}({\text{SN}}_{\text{b,t}},\;{\text{MPV}}_{\text{s,t,T}}) \end{array}$$ (3)

The output G_s_ represents a comprehensive investment recommendation for specific stocks with detailed rationale.

The model’s output includes concise recommendations for Day Trading (Buy First, Sell Later) and Reverse Day Trading (Sell First, Buy Later), along with a clear, step-by-step explanation of the reasoning behind each decision. The terms ‘Day Trading’ and ‘Reverse Day Trading’ are defined within the context of day trading positioning (positive T and reverse T, respectively). We provide an example to show the architecture’s ability to generate interpretable investment recommendations for Chinese banking companies on January 7, 2022, recommending Day Trading (Buy First, Sell Later) operations tomorrow (Appendix Table 2 in S1 File). The table details the intricate signal generation process and illustrates how the model integrates diverse inputs (such as user comments and recent stock trends) to formulate actionable recommendations. This comprehensive approach offers valuable insights for practical decision-making in trading scenarios.

## 3. Result

### 3.1. General comparison

In this experiment, we aimed to compare the effectiveness of different filter-based models in removing redundant data from review datasets. We selected nine banks and evaluated each model based on three key metrics: average excess return, win rate, and profit-loss ratio. We compared the baseline model (which does not incorporate any filters and only use the comment data) with four filter-based models (the filter-1 to filter-4) and the multimodal architecture. Through comparative analysis, we sought to identify which filters demonstrate superior performance in financial analysis. Table 2 presents the average metric values (values in the parentheses represent the gain relative to the baseline model) for the nine banks based on a comparative analysis of the baseline model and four filter-based models (the filter-1 to filter-4 model) across nine banks.

**Table 2:** Average Metric Values for Random Forest Model Comparison.

Models	Excess Return	Win Rate	Profit/Loss Ratio
Baseline	0.0214 (1.00)	0.5740 (1.00)	1.6815 (1.00)
Filter-1	0.0148 (0.69)	0.5614 (0.98)	1.4558 (0.87)
Filter-2	0.0262 (1.22)	0.5973 (1.04)	1.9042 (1.13)
Filter-3	0.0258 (1.21)	0.5995 (1.04)	1.8344 (1.09)
Filter-4	0.0357 (1.67)	0.6244 (1.09)	2.4312 (1.45)
Multimodal	0.0398 (1.86)	0.6325 (1.10)	2.6692 (1.59)

The baseline model yielded an average excess return of 0.0214, a win rate of 0.5740, and a profit-loss ratio of 1.6815, and it was compared with the other models regarding those three metrics. The filter-1 model, however, showed a decline in all performance metrics compared to the baseline model: the excess return dropped to 0.0148 (a decrease of 31%), the win rate fell to 0.5614 (a decrease of 2.2%), and the profit-loss ratio decreased to 1.4558 (a decrease of 13.4%), indicating its ineffectiveness in enhancing returns. The filter-2 model demonstrated notable improvements, with an excess return of 0.0262 (an increase of 22%), a win rate of 0.5973 (an increase of 4%), and a profit-loss ratio of 1.9042 (an increase of 13%). The filter-3 model yielded similar results as the filter-2 model, with an excess return of 0.0258 (an increase of 21%), a win rate of 0.5995 (an increase of 4%), and a profit-loss ratio of 1.8344 (an increase of 9%), suggesting the filter-2 and filter-3 models can improve the baseline model in terms of enhancing returns. Finally, the filter-4 model outperformed the baseline model as well as the other three filter-based models across all indicators, achieving an excess return of 0.0357 (an increase of 67%), a win rate of 0.6244 (an increase of 9%), and a profit-loss ratio of 2.4312 (an increase of 45%).

The multimodal architecture: the multimodal architecture exhibited outstanding performance across all indicators and emerged as the best-performing model: an excess return of 0.0398 (an increase of 86%), a win rate of 0.6325 (an increase of 10%), and a profit-loss ratio of 2.6692 (an increase of 59%) compared to the baseline model. These results show that the multimodal architecture surpasses all other models (the baseline model and the four filter-based models) in all key metrics. From the baseline model to the multimodal architecture, we observed a consistent trend of performance enhancement, which evidenced the value of data filtering and integration. By integrating commentary data with stock volume and price information, the multimodal architecture significantly enhances its ability to generate precise trading signals, optimize timing for buy and sell decisions, and better anticipate market movements. This integration leads to more accurate and reliable investment strategies, ultimately maximizing return on investment. These findings demonstrate the potential of the multimodal architecture to significantly improve the precision and effectiveness of financial analysis and decision-making in trading contexts [43].

### 3.2. Bank-type specific comparison

To further investigate the performance of these filters across different types of banks, we conducted specific comparisons for state-owned banks, joint-venture banks, and local commercial banks separately.

#### 3.2.1. State-owned banks (Appendix Table 3 in S1 File).

Among the three state-owned banks (Agricultural Bank of China, Bank of China, and China Construction Bank), the filter-1 model demonstrated an average excess return of 0.0141 (a decrease of 23%), a win rate of 0.6001 (a decrease of 1%), and a profit-loss ratio of 1.6014 (a decrease of 14%) compared to the baseline model, which suggested that the filter-1 model was inferior to the baseline model. The filter-2 model showed an average excess return rate of 0.0220 (an increase of 21%), a win rate of 0.6353 (an increase of 5%), and a profit-loss ratio of 2.1258 (an increase of 14%). The filter-3 model yielded an excess return rate of 0.0173 (a decrease of 5%), a win rate of 0.6363 (an increase of 5%), and a profit-loss ratio of 1.7735 (a decrease of 4%). The filter-4 model achieved an excess return rate of 0.0267 (an increase of 47%), a win rate of 0.6505 (an increase of 7%), and a profit-loss ratio of 2.5225 (an increase of 36%), holding a clear advantage over the baseline and the other filter-based models. The multimodal architecture performed similarly as the filter-4 model, with an excess return rate of 0.0266 (an increase of 46%), a win rate of 0.6549 (an increase of 8%), and a profit-loss ratio of 2.4578 (an increase of 32%).

#### 3.2.2. Joint-venture banks (Appendix Table 4 in S1 File).

For joint-venture banks (Shanghai Pudong Development Bank, China Minsheng Bank, and China Merchants Bank), the filter-1 model was inferior to the baseline model, with an average excess return rate of 0.0169 (a decrease of 30%), a win rate of 0.5502 (a decrease of 3%), and a profit-loss ratio of 1.4293 (a decrease of 11%). The filter-2 model yielded an excess return rate of 0.0323 (an increase of 33%), a win rate of 0.5897 (an increase of 4%), and a profit-loss ratio of 1.9344 (an increase of 20%), and thus it improved the baseline model. Similarly, the filter-3 model had an excess return rate of 0.0307 (an increase of 27%), a win rate of 0.5802 (an increase of 2%), and a profit-loss ratio of 1.8617 (an increase of 16%). The filter-4 model was significantly better than the other filter-based models with an excess return rate of 0.0441 (an increase of 82%), a win rate of 0.6210 (an increase of 10%), and a profit-loss ratio of 2.5634 (an increase of 60%). The multimodal architecture achieved the best results with an excess return rate of 0.0503 (an increase of 108%), a win rate of 0.6225 (an increase of 10%), and a profit-loss ratio of 2.9795 (an increase of 85%).

#### 3.2.3. Local commercial banks (Appendix Table 5 in S1 File).

Among local commercial banks (Bank of Beijing, Bank of Guiyang, and Bank of Jiangsu), the filter-1 model was again inferior to the baseline model, with an average excess return of 0.0135 (a decrease of 38%), a win rate of 0.5338 (a decrease of 3%), and a profit-loss ratio of 1.3366 (a decrease of 15%). The filter-2 model had an excess return rate of 0.0242 (an increase of 11%), a win rate of 0.5668 (an increase of 3%), and a profit-loss ratio of 1.6525 (an increase of 5%), compared to the baseline model. The filter-3 model yielded an excess return rate of 0.0295 (an increase of 35%), a win rate of 0.5820 (an increase of 6%), and a profit-loss ratio of 1.8680 (an increase of 18%). The filter-4 model outperformed the other filter-based models with an excess return rate of 0.0362 (an increase of 66%), a win rate of 0.6018 (an increase of 10%), and a profit-loss ratio of 2.2078 (an increase of 40%). The multimodal architecture had the best performance, with an excess return rate of 0.0425 (an increase of 95%), a win rate of 0.6200 (an increase of 13%), and a profit-loss ratio of 2.5704 (an increase of 63%).These results underscore effectiveness of filter-based models (particularly the filter-4 model and the multimodal infrastructure) in financial analysis, highlighting their potential to improve decision-making and maximize returns on investment.

## 4. Discussion

### 4.1. Key findings

The comparative analysis of the filter-based models presented in this study reveals important insights into their performances in filtering redundant data and enhancing key financial metrics across various types of banks. By evaluating nine banks of three different types (i.e., state-owned, joint-venture, and local commercial banks), we found that the filter-4 model and the multimodal architecture significantly outperformed the baseline model in terms of excess return, win rate, and profit-loss ratio. The filter-2 and filter-3 models showed modest improvements, while the filter-1 model often performed worse than the baseline model, indicating that simple filtering techniques may not be sufficient [44]. In Appendix Table 6 in S1 File, we present the semantic consistency scores obtained after successive applications of each filtering criterion. The performance comparison indicates that only the filter-4 model, and to some extent the filter-2 model and the filter-3 model, improve the baseline model significantly. The multimodal architecture, due to its use of advanced data integration and analytical techniques, exhibited the most significant gains, highlighting its effectiveness in stock analysis. These results boost our confidence in using large language models (LLMs) and sophisticated filters, underscoring the necessity of implementing such approaches to enhance financial analysis and stock timing decisions.

The analysis (see Appendix Table 3–5 in S1 File) reveals the effects of different filter-based models on financial indicators for banks. The filter-1 model removed reviews shorter than 10 characters and had even weaker performance than the baseline model, indicating that even short reviews can contain valuable information [45]. In contrast, the filter-2 model excluded comments longer than 20 characters, which led to mixed results. While there was a modest improvement in performance metrics (excess return increased to 0.0262 from 0.0214, win rate to 0.5973 from 0.5740, and profit/loss ratio to 1.9042 from 1.6815), the reduction in detail negatively impacted overall data quality. Excluding longer comments might result in loss of valuable information, as longer comments could provide a deeper understanding of market sentiments. This outcome underscores the importance of finding a balance between brevity and detailedness for optimal outcomes. Overly restrictive filters that eliminate longer comments may discard valuable insights, while allowing too much detail can introduce noise and irrelevant information. Striking the right balance is crucial: preserving essential detailed information enhances the richness of the data, while effective filtering reduces noise. Achieving this balance improves the quality of inputs for financial analysis models, leading to better performance and more reliable investment decisions. The filter-3 model utilizes K-means clustering to improve relevance [46], resulting in modest performance gains over the baseline (excess return of 0.0258, win rate of 0.5995, and profit/loss ratio of 1.8344). However, the results indicate that clustering alone may not be sufficient to achieve substantial improvements, highlighting the need for more advanced data refinement techniques.

Filter 4 and the multimodal architecture, both of which employed large language models like GPT-4 for analyzing comments, showed the most significant improvements in performance metrics. The filters 1–3 relied on basic text filtering methods: the filter-1 model removed comments shorter than 10 characters; the filter-2 model excluded comments longer than 20 characters; and the filter-3 model used K-means clustering to select comments. In contrast, the filter-4 model utilized GPT-4 to more effectively filter out low-quality comments. This led to substantial improvements in excess returns, win rates, and profit-loss ratios. To mitigate potential classifier-induced bias, we developed three alternative predictors (LSTM, GBDT, and Transformer-based architectures) that consistently reproduced the observed trend patterns (see Appendix Tables 7–9 in S1 File). The advanced capabilities of GPT-4 allow for a deeper understanding of complex textual data and capture valuable insights that simpler methods missed. The multimodal architecture, which integrates comment analysis with stock price data, achieved the highest performance across all metrics. It’s also important to note that the integration of new textual data with existing financial data created a synergistic effect beyond modeling. This combination enhanced the model’s ability to identify meaningful patterns and trends that neither data source could reveal on its own. By effectively merging qualitative insights from investor comments with quantitative stock information, the multimodal architecture provided a more comprehensive analysis, which led to more accurate predictions and informed decision-making that can significantly improve investment outcomes.

### 4.2. Research contributions

This study makes several key contributions to the field of financial data science: First, we demonstrate the advantage of incorporating investor comment data into financial models. By refining comment datasets using filter-based models, we improved performance metrics such as excess returns and profit-loss ratios. Our findings suggest that both extremely short and long comments can undermine data quality, and thus we confirm the existence of an optimal comment length for financial analysis. Second, the limited improvements observed for the filter-3 model (with the K-means clustering algorithm) indicate the constraints of basic machine learning techniques for data categorization and noise reduction in financial contexts [47–48], and therefore more advanced methods are necessary to significantly enhance performance when processing textual data. Third, the notable advantage of the filter-4 model, which employed the GPT-4 language model, demonstrates the benefit of employing large language models (LLMs) in processing and analyzing textual data for financial applications. Particularly, GPT-4 has notable strength in filtering and interpreting comment data, which can bring substantial improvements in excess returns, win rates, and profit-loss ratios [49]. We also find the multimodal infrastructure which combines diverse data sources (such as filtered comments, stock volume and price data) can create synergistic effects that enhance the model’s ability to identify meaningful patterns and trends, thereby achieving the best performance across all the three metrics. Our findings prove the value of practical applications of sophisticated AI tools in the financial industry.

### 4.3. Limitation

Our study does have several limitations that should be acknowledged. First, our findings are solely on bank stocks, which may not be generalized to other sectors in the stock market. We suggest future research to investigate the performance of our proposed frameworks for non-bank stocks, especially for stocks that are distinct from banks, such as technology companies like Alibaba and Tencent, consumer goods companies like Kweichow Moutai and China Mengniu Dairy, or healthcare firms like Sinopharm and Wuxi Biologics. Second, in calculating excess returns, we did not account for transaction costs, which could have profound impact on the final return [50,51]. This means we might overestimate the profitability of strategies that involve a higher number of trades. Third, though we have included comment data, market quantities & prices, and technical indicators for our analysis, more data (such as macroeconomic data, financial data, and news data) could be used to further boost the predictive performances based on the multimodal infrastructure. Fourth, while our comparative experiments reveal comment data’s predictive significance, the current analysis does not quantify individual feature contributions (e.g., specific technical indicators) or their interactions, which could be further explored through interpretable indices like SHAP in future studies.

## 5. Conclusion

Our research demonstrates that strategically filtering online comments to remove low-quality noise, combined with the analytical power of large language models (LLMs), can enhance stock price prediction accuracy and investment performance. By isolating genuine investor sentiment from irrelevant content, LLMs extract robust sentiment-driven insights which help create a synergistic multimodal framework that amplifies trend and decision-making signals with additional price and volume data—. The fusion of filtered qualitative sentiment and traditional financial metrics improve the predictions, manifested by increases in key performance indicators such as excess returns, win rates, and profit/loss ratios. Our results highlight the transformative role of LLMs in distilling unstructured data into actionable intelligence, offering a scalable AI infrastructure for leveraging online content to refine quantitative investment strategies and optimize portfolio outcomes.

## Supporting information

S1 File Appendix Tables. (DOCX)

S2 File Notes on the figures. (DOCX)

## Abbreviated:

LLMs: Large Language Models
GPT-4: Generative Pre-trained Transformer 4
MA: Moving Average
KDJ: Stochastic Oscillator
MACD: Moving Average Convergence Divergence
BOLL: Bollinger Bands
ABC: Agricultural Bank of China
BOC: Bank of China
CCB: China Construction Bank
SPD BANK: Shanghai Pudong Development Bank
CMBC: China Minsheng Banking Corp
CMB: China Merchant Bank
BOB: Bank of Beijing
GYB: BANK OF Guiyang
JSB: BANK OF Jiangsu
RF: random forest
MCCV: Monte Carlo Cross-Validation.
