# Supplementary material for: Enhancing stock timing predictions based on multimodal architecture: Leveraging large language models (LLMs) for text quality improvement
Source: PLoS One. 2025 Jun 18;20(6):e0326034. doi: 10.1371/journal.pone.0326034 (PMC12176147; doi:10.1371/journal.pone.0326034)
Supplement: S1 File — (DOCX) [file pone.0326034.s001.docx]

**Appendix**

**Appendix Table 1: Daily Comments Summarizer Results for Bank of China on 2022-01-07**

| Comments | remove |
| --- | --- |
| Don't raise it! I still need to save for my retirement. (别涨啊！我还要拿着退休的) | / |
| The magnificent upward trend has begun (波澜壮阔的上升行情开始了) | / |
| Today at 10:46 on the stock market Android version, I would like to ask whether it is better to buy Hong Kong stocks or A shares of Bank of China. (中国人民股神今天10:46股吧Android版请问一下中国银行是买港股好，还是A) | YES |
| After falling for six years, Guiyang Bank began to rise (跌了六年的贵阳银行开始发力上涨) | YES |
| Friendly reminder: once the market starts, there is no looking back. (友情提示行情一启动，就一去不回头了。) | / |
| The first level of pressure volume has been reduced from 570,000 to 300,000, indicating that the chips below 3.07 yuan are locked. (压盘量一档从57万现在减少到30万，说明在3.07元以下的筹码被锁定。这些筹码的) | / |
| Come on, the main force will break through 3.12, and the popularity will rise (主力加油， 突破3.12，人气就上来了) | / |
| 3.10 is half done, time-consuming and labor-intensive, and I earned nothing (3.10出一半，耗时耗力的东西，赚了个寂寞) | / |
| Is there anyone who buys Bank of China hoping to make money by seeing its stock price rise? (买中行有指着股价上涨挣钱的吗？) | YES |
| Ahhh, it's sold too early. (啊啊啊卖早了) | / |
| The main force has been shipped and will be available soon. (主力出货了，很快就会下来的) | YES |
| Is it better for Bank of China to buy Hong Kong stocks or A shares? Please give me some advice, experts. (中国银行买港股好，还是A股，各位大佬请指教。) | YES |
| On January 6, Bank of China received an increase of 2.751 million shares through Shanghai Stock Connect (中国银行01月06日获沪股通增持275.1万股) | / |
| Bank of China: Net financing purchase of 12.0502 million yuan, financing balance of 3.489 billion yuan (01-06) (中国银行：融资净买入1205.02万元，融资余额34.89亿元（01-06）) | YES |
| The fight for power always takes place in the wind and rain. Believe that sticking power will bring you a rainbow. Stick all the way until it returns to zero. Only then will the flying paper be useful. (抢权总在风雨中～相信贴权有采虹～一路贴到归零终～那张纸飞飞才有用) | YES |

**Appendix Table 2：Bank of China Generated Signal and Explanation (2022-01-07)**

| Decision | Explanation |
| --- | --- |
| Day Trading | Based on the provided data, our recommended strategy is Day Trading (Buy First, Sell Later). The rationale for this recommendation is as follows:  1. **Analysis from Comments Data**: The sentiment analysis of comments indicates that individual investors are highly optimistic about the future market performance of Beijing Bank. This positive sentiment suggests strong investor confidence, which can be a driving factor for short-term price movements.  2. **Analysis from Stock Data**: The technical analysis of the stock data reveals a bullish trend. The stock has recently experienced a moving average crossover, indicating upward momentum. This crossover is a key technical indicator that often precedes price increases, making it a favorable condition for executing a buy-first, sell-later strategy.  These analyses collectively suggest that engaging in day trading for Beijing Bank stock could capitalize on the current positive sentiment and technical indicators, potentially leading to profitable intraday trading opportunities. |

**Appendix Table 3:** **Model Performance** **for Nation-Owned Banks**

| Models | Bank Names | Excess Return | Win Rate | Profit/Loss Ratio |
| --- | --- | --- | --- | --- |
| baseline | Agricultural Bank of China (ABC) | 0.0187 | 0.6428 | 2.1705 |
|  | Bank of China (BOC) | 0.0091 | 0.5786 | 1.5419 |
|  | China Construction Bank(CCB) | 0.0268 | 0.6008 | 1.8578 |
|  | Average value | 0.0182 (1.00) | 0.6074 (1.00) | 1.8567 (1.00) |
| filter-1 | Agricultural Bank of China (ABC) | 0.0112 | 0.6115 | 1.5627 |
|  | Bank of China (BOC) | 0.0091 | 0.5938 | 1.5544 |
|  | China Construction Bank(CCB) | 0.0221 | 0.5950 | 1.6872 |
|  | Average value | 0.0141 (0.77) | 0.6001 (0.99) | 1.6014 (0.86) |
| filter-2 | Agricultural Bank of China (ABC) | 0.0200 | 0.6593 | 2.2457 |
|  | Bank of China (BOC) | 0.0144 | 0.6230 | 2.0466 |
|  | China Construction Bank(CCB) | 0.0317 | 0.6235 | 2.0852 |
|  | Average value | 0.0220 (1.21) | 0.6353 (1.05) | 2.1258 (1.14) |
| filter-3 | Agricultural Bank of China (ABC) | 0.0162 | 0.6671 | 1.9064 |
|  | Bank of China (BOC) | 0.0097 | 0.6066 | 1.5879 |
|  | China Construction Bank(CCB) | 0.0261 | 0.6351 | 1.8261 |
|  | Average value | 0.0173 (0.95) | 0.6363 (1.05) | 1.7735 (0.96) |
| filter-4 | Agricultural Bank of China (ABC) | 0.0259 | 0.6616 | 2.9536 |
|  | Bank of China (BOC) | 0.0139 | 0.6123 | 1.9759 |
|  | China Construction Bank(CCB) | 0.0403 | 0.6777 | 2.6381 |
|  | Average value | 0.0267 (1.47) | 0.6505 (1.07) | 2.5225 (1.36) |
| multimodal | Agricultural Bank of China (ABC) | 0.0245 | 0.6613 | 2.7000 |
|  | Bank of China (BOC) | 0.0150 | 0.6390 | 2.0592 |
|  | China Construction Bank(CCB) | 0.0402 | 0.6643 | 2.6142 |
|  | Average value | 0.0266 (1.46) | 0.6549 (1.08) | 2.4578 (1.32) |

**Appendix Table 4: Model Performance for Joint-stock Commercial Banks**

| Models | Bank Names | Excess Return | Win Rate | Profit/Loss Ratio |
| --- | --- | --- | --- | --- |
| baseline | Shanghai Pudong Development Bank(SPD BANK) | 0.0160 | 0.5539 | 1.5105 |
|  | China Minsheng Banking Corp(CMBC) | 0.0087 | 0.5383 | 1.4176 |
|  | China Merchant Bank(CMB) | 0.0480 | 0.6070 | 1.8907 |
|  | Average value | 0.0242 (1.00) | 0.5664 (1.00) | 1.6063 (1.00) |
| filter-1 | Shanghai Pudong Development Bank(SPD BANK) | 0.0092 | 0.5313 | 1.2807 |
|  | China Minsheng Banking Corp(CMBC) | 0.0100 | 0.5354 | 1.4888 |
|  | China Merchant Bank(CMB) | 0.0315 | 0.5840 | 1.5183 |
|  | Average value | 0.0169 (0.70) | 0.5502 (0.97) | 1.4293 (0.89) |
| filter-2 | Shanghai Pudong Development Bank(SPD BANK) | 0.0230 | 0.5753 | 1.8224 |
|  | China Minsheng Banking Corp(CMBC) | 0.0125 | 0.5568 | 1.6345 |
|  | China Merchant Bank(CMB) | 0.0613 | 0.6370 | 2.3462 |
|  | Average value | 0.0323 (1.33) | 0.5897 (1.04) | 1.9344 (1.20) |
| filter-3 | Shanghai Pudong Development Bank(SPD BANK) | 0.0151 | 0.5617 | 1.4826 |
|  | China Minsheng Banking Corp(CMBC) | 0.0132 | 0.5560 | 1.6940 |
|  | China Merchant Bank(CMB) | 0.0639 | 0.6230 | 2.4086 |
|  | Average value | 0.0307 (1.27) | 0.5802 (1.02) | 1.8617 (1.16) |
| filter-4 | Shanghai Pudong Development Bank(SPD BANK) | 0.0258 | 0.5930 | 1.9869 |
|  | China Minsheng Banking Corp(CMBC) | 0.0194 | 0.5897 | 2.1836 |
|  | China Merchant Bank(CMB) | 0.0872 | 0.6804 | 3.5197 |
|  | Average value | 0.0441 (1.82) | 0.6210 (1.10) | 2.5634 (1.60) |
| multimodal | Shanghai Pudong Development Bank(SPD BANK) | 0.0308 | 0.5657 | 2.2473 |
|  | China Minsheng Banking Corp(CMBC) | 0.0191 | 0.6017 | 2.1475 |
|  | China Merchant Bank(CMB) | 0.1009 | 0.7000 | 4.5437 |
|  | Average value | 0.0503 (2.08) | 0.6225 (1.10) | 2.9795 (1.85) |

**Appendix Table 5: Model Performance for Local Commercial Banks**

| Models | Bank Names | Excess Return | Win Rate | Profit/Loss Ratio |
| --- | --- | --- | --- | --- |
| baseline | Bank of Beijing(BOB) | 0.0057 | 0.5263 | 1.2537 |
|  | BANK OF GUIYANG(GYB) | 0.0179 | 0.5337 | 1.6652 |
|  | BANK OF JIANGSU(JSB) | 0.0419 | 0.5843 | 1.8252 |
|  | Average value | 0.0218 (1.00) | 0.5481 (1.00) | 1.5814 (1.00) |
| filter-1 | Bank of Beijing(BOB) | 0.0048 | 0.5176 | 1.2083 |
|  | BANK OF GUIYANG(GYB) | 0.0102 | 0.5172 | 1.3459 |
|  | BANK OF JIANGSU(JSB) | 0.0255 | 0.5665 | 1.4555 |
|  | Average value | 0.0135 (0.62) | 0.5338 (0.97) | 1.3366 (0.85) |
| filter-2 | Bank of Beijing(BOB) | 0.0067 | 0.5395 | 1.3045 |
|  | BANK OF GUIYANG(GYB) | 0.0152 | 0.5370 | 1.5331 |
|  | BANK OF JIANGSU(JSB) | 0.0508 | 0.6239 | 2.1198 |
|  | Average value | 0.0242 (1.11) | 0.5668 (1.03) | 1.6525 (1.05) |
| filter-3 | Bank of Beijing(BOB) | 0.0103 | 0.5617 | 1.5007 |
|  | BANK OF GUIYANG(GYB) | 0.0198 | 0.5504 | 1.7543 |
|  | BANK OF JIANGSU(JSB) | 0.0585 | 0.6339 | 2.3490 |
|  | Average value | 0.0295 (1.35) | 0.5820 (1.06) | 1.8680 (1.18) |
| filter-4 | Bank of Beijing(BOB) | 0.0130 | 0.5897 | 1.6761 |
|  | BANK OF GUIYANG(GYB) | 0.0218 | 0.5697 | 1.8495 |
|  | BANK OF JIANGSU(JSB) | 0.0738 | 0.6459 | 3.0979 |
|  | Average value | 0.0362 (1.66) | 0.6018 (1.10) | 2.2078 (1.40) |
| multimodal | Bank of Beijing(BOB) | 0.0154 | 0.6088 | 1.8494 |
|  | BANK OF GUIYANG(GYB) | 0.0280 | 0.5943 | 2.1754 |
|  | BANK OF JIANGSU(JSB) | 0.0840 | 0.6570 | 3.6864 |
|  | Average value | 0.0425 (1.95) | 0.6200 (1.13) | 2.5704 (1.63) |

**Appendix Table 6: Performance Comparison of Filtering Algorithms on Semantic Consistency Metrics^[[1]](#footnote-1)^**

| Bank Names | Baseline | Filter-1 | Filter-2 | Filter-3 | Filter-4 |
| --- | --- | --- | --- | --- | --- |
| Bank of Beijing(BOB) | 0.5893 | 0.5873 | 0.6244 | 0.6345 | 0.6691 |
| BANK OF GUIYANG(GYB) | 0.6645 | 0.6625 | 0.6734 | 0.6862 | 0.6745 |
| China Construction Bank(CCB) | 0.6045 | 0.5983 | 0.6290 | 0.6507 | 0.6696 |
| BANK OF JIANGSU(JSB) | 0.6205 | 0.6188 | 0.6596 | 0.6757 | 0.7115 |
| China Minsheng Banking Corp(CMBC) | 0.5970 | 0.5940 | 0.6188 | 0.6455 | 0.6804 |
| Agricultural Bank of China (ABC) | 0.6039 | 0.6017 | 0.6267 | 0.6427 | 0.6699 |
| Shanghai Pudong Development Bank(SPD BANK) | 0.6308 | 0.6294 | 0.6537 | 0.6770 | 0.6698 |
| China Merchant Bank(CMB) | 0.5986 | 0.5969 | 0.6218 | 0.6507 | 0.6604 |
| Bank of China (BOC) | 0.6125 | 0.6082 | 0.6432 | 0.6530 | 0.7072 |
| Average value | 0.6135 | 0.6108 | 0.6389 | 0.6573 | 0.6792 |

**Appendix Table 7: Average Metric Values for GBDT Model Comparison^[[2]](#footnote-2)^**

| Models | Excess Return | Win Rate | Profit/Loss Ratio |
| --- | --- | --- | --- |
| Baseline | 0.0373 | 0.6072 | 2.3569 |
| Filter-1 | 0.0230 | 0.5735 | 1.6647 |
| Filter-2 | 0.0435 | 0.6520 | 2.7854 |
| Filter-3 | 0.0437 | 0.6464 | 2.8009 |
| Filter-4 | 0.0481 | 0.6600 | 3.1783 |
| Multimodal | 0.0492 | 0.6600 | 3.2393 |

**Appendix Table 8: Average Metric Values for LSTM Model Comparison^[[3]](#footnote-3)^**

| Models | Excess Return | Win Rate | Profit/Loss Ratio |
| --- | --- | --- | --- |
| Baseline | 0.0021 | 0.5578 | 1.0458 |
| Filter-1 | 0.0020 | 0.5578 | 1.0452 |
| Filter-2 | 0.0021 | 0.5578 | 1.0458 |
| Filter-3 | 0.0143 | 0.5793 | 1.5673 |
| Filter-4 | 0.0467 | 0.6447 | 3.0834 |
| Multimodal | 0.0491 | 0.6575 | 3.2382 |

**Appendix Table 9: Average Metric Values for Transformer-based Model Comparison^[[4]](#footnote-4)^**

| Models | Excess Return | Win Rate | Profit/Loss Ratio |
| --- | --- | --- | --- |
| Baseline | 0.0285 | 0.6155 | 2.1053 |
| Filter-1 | 0.0218 | 0.5697 | 1.7213 |
| Filter-2 | 0.0374 | 0.6282 | 2.6313 |
| Filter-3 | 0.0429 | 0.6331 | 2.7987 |
| Filter-4 | 0.0481 | 0.6520 | 3.2379 |
| Multimodal | 0.0508 | 0.6682 | 3.4117 |

1. Semantic consistency scores were calculated using the sentiment analysis module of the SnowNLP package. Sentiment polarity scores for textual comments were derived from the sentiments function, where a score above 0.5 was classified as a positive comment, and a score equal to or below 0.5 as negative. To quantify emotional coherence after filtering, the sentiment consistency ratio for each filter was computed as: Ratio = max(N_positive, N_negative) / N_total , where N_positive and N_negative represent the counts of positive and negative comments, respectively, and N_total denotes the total number of comments. This metric enables systematic comparison of sentiment alignment across different filtering algorithms. [↑](#footnote-ref-1)
2. The value in parentheses represents the gain relative to the baseline model. The GBDT model was implemented with n_estimators=50 and learning_rate=0.1 while preserving all other default hyperparameter configurations specified in the scikit-learn library. [↑](#footnote-ref-2)
3. The value in parentheses represents the gain relative to the baseline model. The LSTM model was implemented with hidden_size=64 and num_layers=2, employing the CrossEntropyLoss function and the Adam optimizer (learning_rate=0.001 with L2 regularization weight_decay=1e-4). The training configuration utilized a batch_size=16 and 10 training epochs (with 10 internal iterations per epoch), while applying dropout=0.2 and gradient clipping (max_norm=1.0); all other hyperparameters retained their default configurations as per the PyTorch library. [↑](#footnote-ref-3)
4. The value in parentheses represents the gain relative to the baseline model. The Transformer-based model employs a classical encoder architecture with the following core configurations: (1) A 2-layer stacked Transformer encoder, where each layer integrates 4-head scaled dot-product attention mechanisms and 128-dimensional feed-forward networks; (2) Input features are linearly projected into a 64-dimensional space through an embedding layer, followed by sinusoidal/cosine positional encoding; (3) Binary classification is achieved via first-element sequence pooling and a dual-layer classification head (64 → ReLU activation → Dropout (p=0.1) → 2-output linear projection). The model is trained using the Adam optimizer (learning rate = 0.001) for 10 epochs, with each epoch comprising 10 complete data passes through a batch size of 32 and default L2 regularization. Input data is processed as single-sample sequences (seq_len=1), where raw features are scaled by the square root of the model dimension prior to embedding layer projection. [↑](#footnote-ref-4)
