# Supplementary material for: Enhancing stock timing predictions based on multimodal architecture: Leveraging large language models (LLMs) for text quality improvement
Source: PLoS One. 2025 Jun 18;20(6):e0326034. doi: 10.1371/journal.pone.0326034 (PMC12176147; doi:10.1371/journal.pone.0326034)

**Fig 1: Design and Evaluation Workflow of Data Screening Filters**


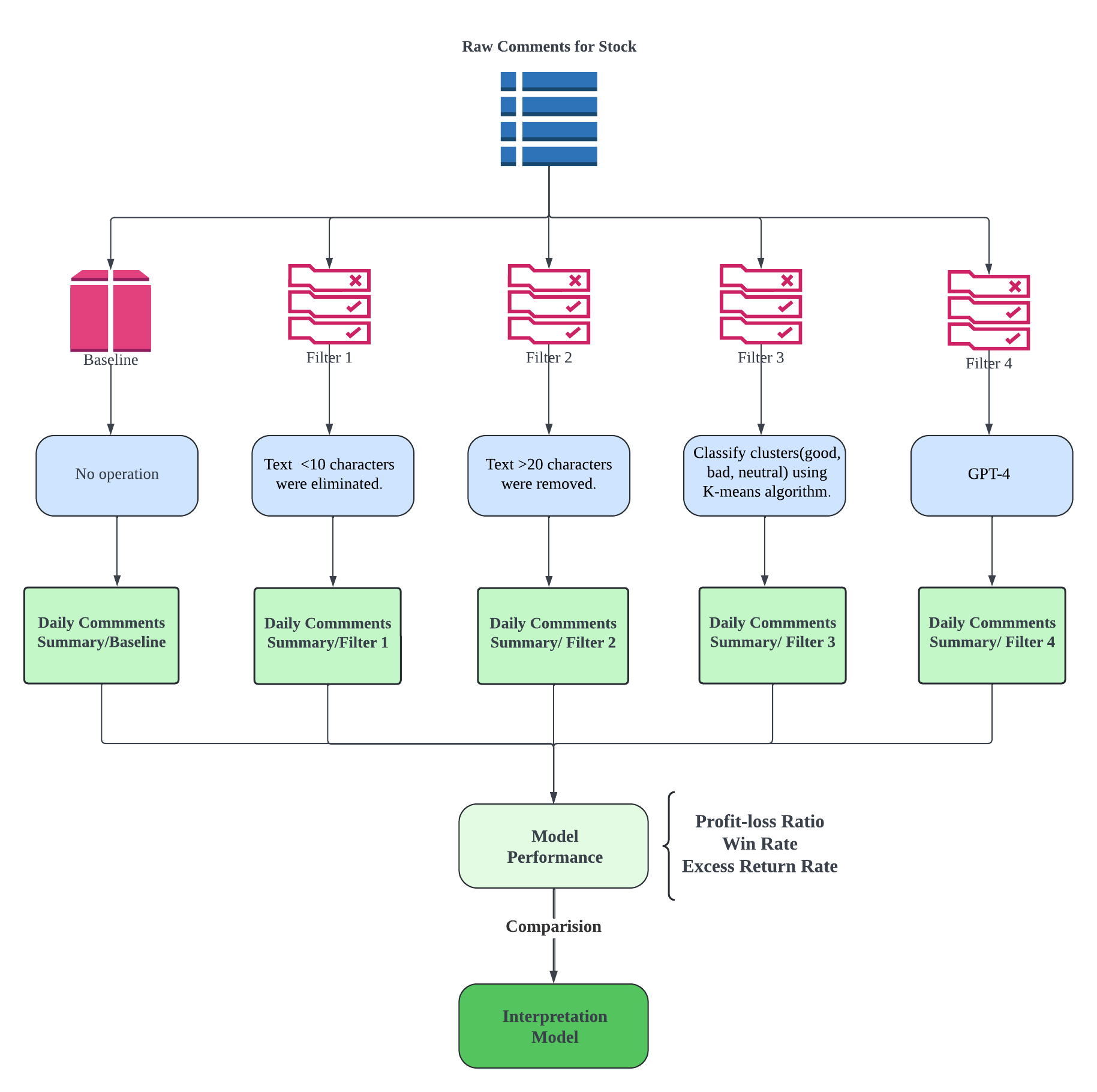


Notes:

Baseline: The baseline approach retains raw comments without preprocessing, preserving the complete dataset's original distribution. This includes all textual noise (e.g., typos, slang, emojis) and unfiltered content, serving as a control group to quantify the value added by subsequent preprocessing stages. The Filter-1 Model: We implement a character-based noise reduction protocol, systematically removing comments shorter than 10 characters (e.g., "HODL," "To the moon!"). This eliminates about 23% of the dataset statistically dominated by non-actionable hype (validated by χ² tests, *p<0.001*). The Filter-2 Model: To counter verbosity-induced signal distortion, we exclude comments exceeding 20 characters—a boundary where relevance decays exponentially. This removes rambling narratives (e.g., multi-sentence personal anecdotes) while preserving concise technical signals ("MACD crossover at 150 yuan"). The Filter-3 Model: We apply the K-means clustering algorithm to classify raw comments into three sentiment-based clusters (good, bad, and neutral), leveraging TF-IDF vectorization to quantify textual relevance. The optimal cluster count (k=3) is validated using silhouette analysis, ensuring distinct separation of sentiment patterns. The cluster with the highest comment density—often representing market consensus—is selected as the final dataset. This majority-driven selection minimizes outlier noise while preserving statistically significant trends, enabling refined analysis of dominant investor sentiment aligned with price movements. The Filter-4 Model: We use the GPT-4 large language model API to perform semantic triage on comments, integrating context-aware redundancy removal (e.g., eliminating duplicate opinions) and logical consistency checks (e.g., flagging contradictory statements like "bullish fundamentals" vs. "immediate sell signal"). GPT-4’s zero-shot classification capability further categorizes comments into actionable, speculative, or irrelevant tiers based on predefined financial criteria. By filtering out non-actionable noise (e.g., memes, off-topic remarks) and retaining comments with quantifiable insights (e.g., mentions of P/E ratios, earnings forecasts), the model elevates dataset quality, directly enhancing the accuracy of downstream metrics like excess return rate and profit-loss ratio. The implementation deploys model="gpt-4.1" with controlled generation parameters (temperature=0.2, max_tokens=1000, frequency_penalty=0.0), system prompts is "You are a helpful assistant for text processing"，the formal definition of user prompts is documented in Notes (1): Rule 4 - comment remove prompt template .

**Fig 2: The Conceptual Framework of the multimodal architecture for optimizing the excess return rate.**


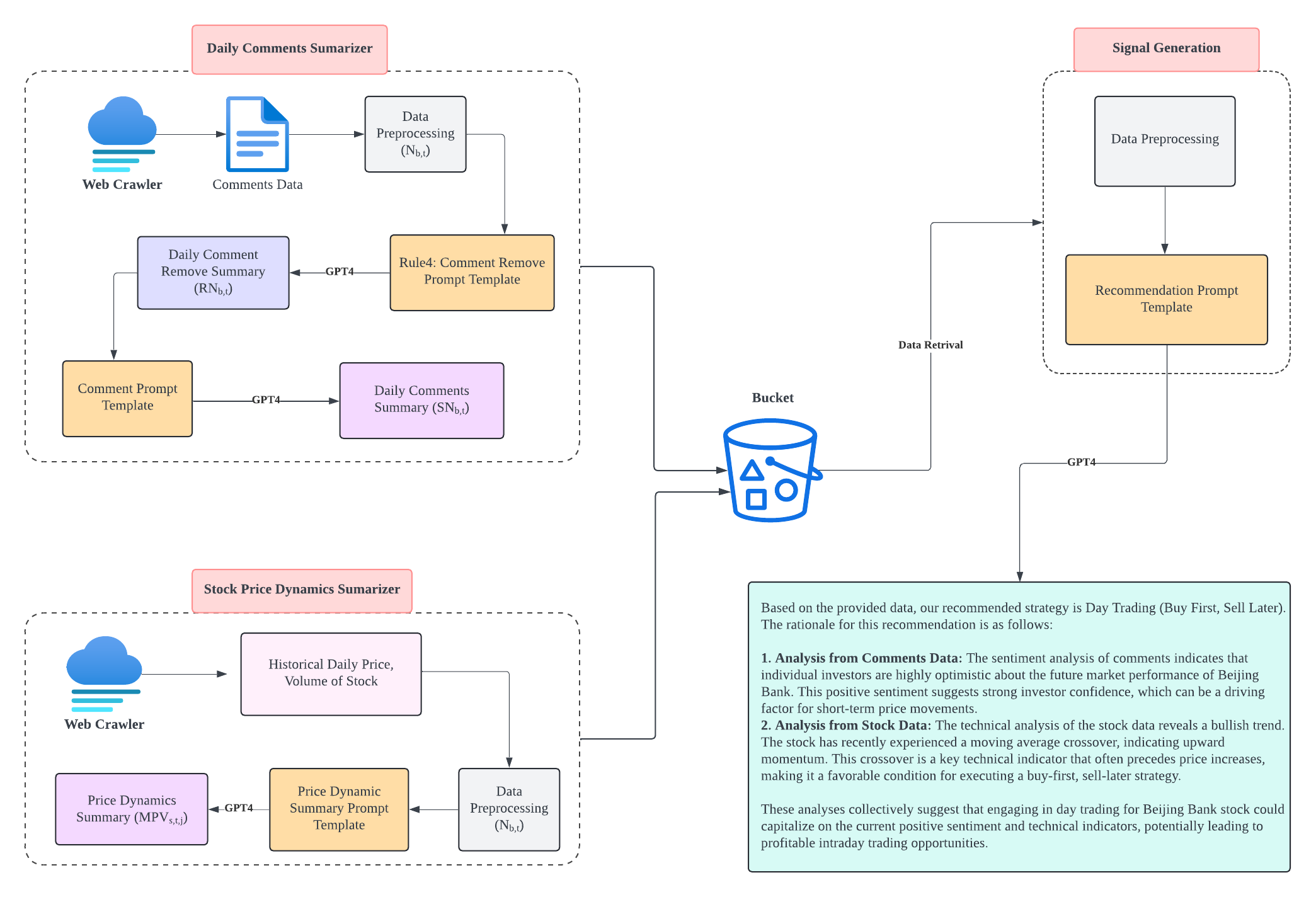


Notes:

1. Rule4: comment remove prompt template

As a professional financial data cleansing specialist, execute the following tasks:

[Task Instruction]

Perform contextual redundancy elimination and logical consistency verification with requirements:

1. Redundancy Filtering:

• Remove comments with ≥3 consecutive repeated characters (e.g. "gogogo！！!" → marked as spam)

• Eliminate content containing advertising identifiers (regex: /【.*】|Click to download | Get red envelope/)

2. Logical Verification:

• Flag semantically contradictory statements (e.g. co-occurrence of "Solid growth in fundamentals" and "Strong short-term bearish")

• Identify irregular comments with >60% non-Chinese characters

[Output Format]

{ valid_comment: str, conflict_flags: str, reason: str }

1. comment prompt template

As a professional sentiment analyst, process requirements:

[Data Processing Specifications]

Aggregate raw comments for {stock_code} on {date} into structured data:

1. Sentiment Analysis:

• Output polarity labels (positive/neutral/negative)

• Calculate sentiment intensity scores (1-5 scale, 5=most intense)

2. Keyword Extraction:

• Institutional mentions: count frequency of "Main force/institutional/northbound" keywords

• Hotspot identification: extract industry terms with >2% occurrence frequency

[Output Format]:

{ date: "YYYY-MM-DD", sentiment_clsss: str, data: [str] }

1. price dynamic summary prompt template

As a quantitative strategy researcher, perform analysis:

[Technical Indicator Generation Rules]

Input: {stock_code} OHLCV data (daily) for {date}

Processing Pipeline:

1. Data Cleaning:

• Exclude call auction period (09:15-09:25)

• Repair outliers (activate linear interpolation when price fluctuation >5%)

2. Feature Calculation:

• MA indicators: MA5/MA20 crossover status (Golden Cross/Death Cross/None)

• MACD indicators: macd

• KDJ indicators: kdj

[Output Format]:

{ date: "YYYY-MM-DD", ma_cross: str, macd: str, kdj: str, OHLCV: {open: float, high: float, low: float, close: float, volume: float} }

1. recommendation prompt template

As an algorithmic trading strategist, execute multimodal decision-making:

[Multimodal Decision Instruction]

Generate next-day price movement prediction based on:

• Comment data

• Price dynamics

• Technical signals

[Generation Requirements]

1. Prediction Conclusion:

• >60% upside probability → "Strong Buy"

• 40%-60% → "Neutral"

• <40% → "Sell Warning"

2. Rationale Specification:

• Must include synergy analysis between sentiment and technical aspects

[Output Format]:

{ prediction: str, confidence: float, rationale: str, action: str }

**Fig 3. Architectural Breakdown of the Daily Comments Summarizer Module**


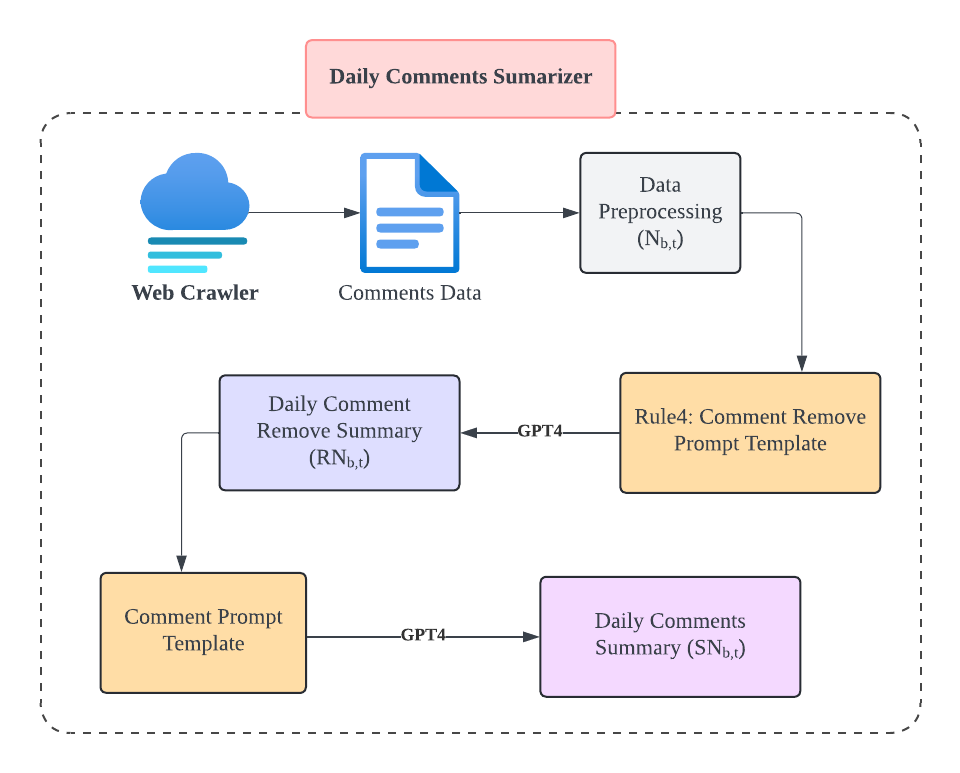


**Fig 4. Architectural Breakdown of the Stock Price Dynamic Summarizer Module**


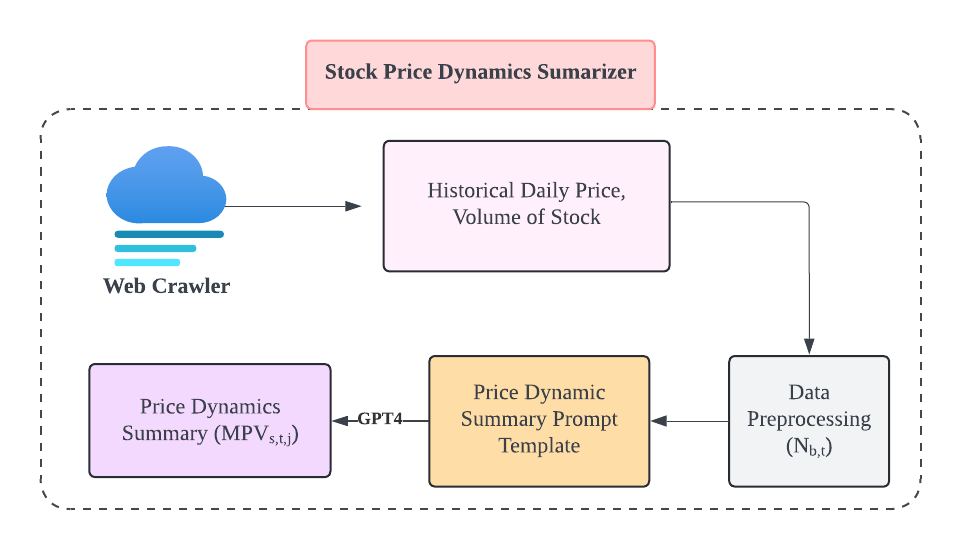


**Fig 5. Architectural Breakdown of the Signal Generation Module**


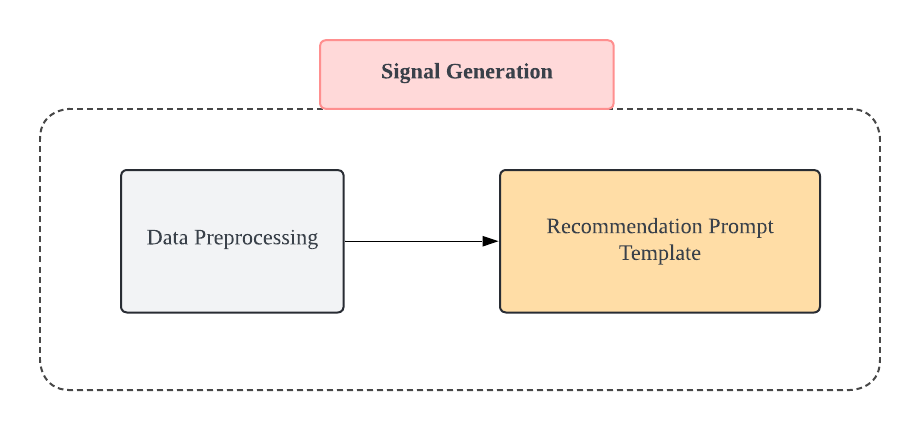

Supplement: S2 File — (DOCX) [file pone.0326034.s002.docx]
